# A comparative study of unintentional injuries among schooling left-behind, migrant and residential children in China

**DOI:** 10.1186/s12939-018-0767-3

**Published:** 2018-04-23

**Authors:** Hongwei Hu, Jiamin Gao, Haochen Jiang, Pingnan Xing

**Affiliations:** 1 School of Public Administration and Policy, Renmin University of China, No. 59, Zhongguancun Street, Haidian District, Beijing, 100872 People’s Republic of China; 2 Guanghua School of Management, Peking University, No.5, Yiheyuan Road, Haidian District, Beijing, 100871 People’s Republic of China; 3 School of Humanities and Social Sciences, North China Electric Power University, No.689, Huadian Road, Lianchi District, Baoding, Hebei 071003 People’s Republic of China

**Keywords:** Unintentional injury, Left-behind children, Migrant children, Family migration, Risk factor

## Abstract

**Background:**

Children who suffer from parental migration have been manifested to exhibit physical and mental impairments at higher rates. This current study aims to explore unintentional injury disparity among schooling left-behind children, migrant children and residential children in China, and to examine the risk factors of unintentional injury among the three types of children based on a multi-level system framework. This study will fill the gaps of this topic for China and contribute to the world literature in the context of countries with frequent population migration.

**Methods:**

Data for 4479 children aged 6–16 of a representative population sample were obtained from a survey conducted in China in 2017. Child’s unintentional injury in this survey was measured based on the definition and classification of ICD-10. Descriptive analysis, multivariable logistic regression and zero-inflated negative binomial regression were employed in this study.

**Results:**

Left-behind children showed higher prevalence of total unintentional injury than migrant and residential children, as well as in 14 specific unintentional injuries. There was a statistical difference between left-behind and residential children’s unintentional injuries, but no significant difference was found between migrant and residential children. Results also indicated that both individual and environmental factors constructed as a multi-level system were associated with children’s unintentional injuries.

**Conclusions:**

Family migration may have contributed to the increased unintentional injury risks among children. Left-behind children were more vulnerable to suffer from unintentional injuries than migrant and residential children, and specific attentions should be paid to unique group of children, especially the left-behind children. Given the importance and serious consequences of children’s unintentional injuries, the findings may provide implications for necessary intervention.

## Background

Child injury, a leading cause of deaths and disabilities among children throughout the world, has become a major public health concern in the globe [1], and unintentional injury accounts for the largest proportion among all children injuries. Unintentional injuries happened to children through lacking of reasonable care or failure to protect [2], which can be classified as traffic injuries, falls, burns, poisonings and drowning. Approximately 2000 families were affected by children unintentional injuries per day [1]. In low-and middle-income countries, unintentional injuries of children accounted for over 7% of total deaths and over 9% of total disability-adjusted life-years [3]. Unintentional injury can lead to irreversible consequences for most children and untreated accidental injury is associated with numerous adverse outcomes, including death, disability and increasing demand for hospital care [4]. With the deepening on urbanization and industrialization, as well as changes for environments, children are more likely to be exposed to unintentional injuries.

Plenty of evidences have proved that childhood unintentional injury is associated with broad factors which are constructed as a multi-level system. Individual characteristics and the circumstances factors including family, school and society in which they live, vary greatly among children and are related to the types, causes and patterns of child unintentional injury. In particular, sociodemographic factors, family and school circumstance factors are the main factors that have influence on child unintentional injury. Studies have indicated that children’s physical and cognitive abilities, degrees of dependence as well as activity abilities developed by age, made them face with different injury risks [5]. Generally speaking, boys are more frequent to experience injuries, but a study in South Asia indicated that girls were easier to suffer from unintentional injury, especially for burns due to cooking [6]. With regard to family level, most of child’s accidental injury occurs at home and in the surrounding environments. Family factors, such as family structure (i.e., single parenting, number of children and family relationships) and household socioeconomic status (i.e., maternal education, family income and parental occupation status), exert impacts on child’s unintentional injury risks [7]. Besides, studies pointed out that schools were another environment where child injury frequently occurred [8]. Children with poor school performance and learning pressure can lead to negative self-recognition which may increase the risks for unintentional injury [9]. As for residence, a study indicated that children’s unintentional injuries in China can be explained in parts by urban-rural disparity, especially for drowning, road traffic injuries and accidental asphyxiation [10].

In China, child mortality rate caused by unintentional injury was 26.1% in 2008 [11]. Unintentional injury remained the leading cause on deaths of children aged 0–14 [12]. In addition, non-fatal injuries caused by accidents were ranked as the major cause of morbidity, which lead to heavy disease burden [13]. Especially, it is important to note that left-behind and migrant children who are exposed to parental migration have been shown to suffer from unintentional injuries at higher rates. Since the early 1990s, China has been undergoing rapid economic and social changes which gives rise to a large scale of population migration. In this social context, with many adults moving from villages to cities for better paying jobs, dramatically increasing amount of left-behind children and migrant children appears in Chinese society. The amount of left-behind children whose parent(s) migrated to cities reached 61.02 million in China in 2010, while migrant children reached 35.81 million [14]. Studies demonstrated that compared to residential children, left-behind and migrant children exhibited higher level of emotional problems and unhealthy behaviors including smoking or alcohol abuse, which may increase the risks for unintentional injuries [15]. Besides, a study in Guangzhou indicated that the incidence rate of injury death was significantly higher among migrant children than residential children [16].

Left-behind and migrant children may experience changes of family structure and circumstances. Absence of parental care or having difficulty in adapting to new life/environment make left-behind children and migrant children more vulnerable for unintentional injury. A fair proportion of studies deepen and broaden the understandings of individual and environmental effects on child unintentional injury [17], however, there are several limitations in the previous literature. First, the number of studies regarding unintentional injuries of left-behind children or migrant children in China were limited; second, there were insufficient studies concerning on comparison of unintentional injuries among the three types of children; third, the sample sizes of left-behind children or migrant children in previous studies about unintentional injuries were small; last but not the least, most of previous literatures failed to analyze the associated factors of unintentional injuries in the perspective of multi-level system, resulting in over emphasis on specific dimension rather than a whole system.

This present study focuses on two research questions. First, it examines the unintentional injury prevalence of migrant and left-behind children, compared to the residential children in China. Second, given the potential impacts of individual characteristics and family, school and residential environments on child development, this study discusses the association between the multi-level risk factors and unintentional injuries of children, based on the ecological theoretical framework. Data used in this study were from a survey which was designed to explore the unintentional injury outcomes of migrant, left-behind and residential children in Chongqing, one typical city with a large scale of immigration and emigration in China. Given the importance and serious consequences of children’s unintentional injuries, we hope the findings may contribute to a clearer understanding of children unintentional injuries and provide implications for necessary intervention in China. This study will fill the gaps in the comparison of unintentional injuries among three different types of children in China, examine the risk factors on unintentional injuries in a multi-level system and contribute to the world literature in the context of countries with frequent population migration.

## Methods

### Theoretical basis and hypothesis

Ecological model which rooted in Urie Bronfenbrenner’s concept of childhood development was found to be applied in the association between environment and child health, as well as well-being [18, 19]. It provides a broad context for understanding how environments be in association with physical health of children as contextual developmental factors. The ecological environment, as a set of nested structures, is consisted of five dimensions including micro-system, meso-system, exo-system, macro-system and chrono-system [20]. On the basic of the five dimensions in ecological model, this study selected control variables from the aspects of child individual characteristics, family, school and residence environments.

Based on the ecological model’s framework, we suppose that unintentional injury outcome of children in China is a result of multi-level system, which is related to both individual factors and contextual environments. Upon this assumption, we propose the following hypothesis:

First, compared to residential children, migrant and left-behind children have higher prevalence of unintentional injury due to the change of ecological environment system they lived in;

Second, the risks of child unintentional injury are not only associated with personal characteristics, but also correlated with the direct and indirect environments they live in.

### Data

Data were obtained from the Survey on Physical and Mental Health of Children, a school-based survey on children health outcomes which conducted in Chongqing, China, from September to October in 2017, by Chongqing Education Bureau and other educational institutions. Using the method of multi-stage stratified sampling, 30 schools from 9 counties were selected; one class was chosen randomly in each grade within selected schools. Parents or guardians of the children in selected classes were contacted by investigators to ask permission to complete the interviews. Children’s information was collected by asking their parents, either father or mother, or other guardians to fill out the survey questionnaires. The original selected sample included 4500 children aged 6–16, and 4479 participated in the survey with complete information on key variables.

### Measures

The definition and classification of unintentional injury in this study was in accordance with the codes for external causes of injury from the International Statistical Classification of Diseases and Related Health Problems, 10th Revision (ICD-10) [21]. These causes were also among the fundamental subsets of unintentional injury recommended in the Chinese CDC-National Injury Surveillance System(NISS), Work Manual of National Injury Surveillance System [22].

On a reliable basis of ICD-10, this study retrieved all the relevant researches on child unintentional injury in recent decades in the CNKI (China National Knowledge Infrastructure), the largest Chinese electronic database including academic papers, newspapers and other literature, finding the actual situation of child unintentional injury in China. According to the searching results, 14 kinds of unintentional injury from ICD-10, containing vehicle and traffic injuries, falls and struck by thrown, projected or falling objects, were regarded as representative types of unintentional injury Chinese children suffered from. The outcome measures were defined as a binary variable-- whether an unintentional injury from the 14 selected types of unintentional injury occurred, as well as a count variable-- the number of types on accidental injury that children suffered from. The unintentional injury was reported by child’s parents or guardians in response to the following question: “Have your children ever suffered from accidental injury in the past one year?” If participants answered “yes” during the survey, investigators would continue to ask what kind of injury and how many types of injury that the child had experienced within the 14 selected types of unintentional injury.

Child category was employed as the main independent variable in this study. Residential children were defined as children that were registered and living with parents in Chongqing. Migrant children were those who did not register but living with parent(s) in Chongqing. Children registered and were living in Chongqing, but both or at least one parent migrated to work elsewhere for more than 6 months were defined as left-behind children.

Based on ecological theoretical framework, other independent variables in this study included multi-level variables: children’s sociodemographic and health characteristics, parental and household factors, school situations and, residence. Sociodemographic and health characteristic variables consisted of gender (0 = girl and 1 = boy), age (0 = 6–11 years old and 1 = 12–16 years old), physical health conditions (0 = good and 1 = fair), school academic achievement (0 = good, 1 = fair and 2 = poor), the only-child in family (0 = no and 1 = yes). Parental and household factors referred to household income level (0 = high, 1 = medium and 2 = low), parental marital status (0 = unmarried and 1 = married), maternal education attainment (0 = elementary school and below, 1 = middle school and above), family conflicts (0 = frequent and 1 = seldom or never). We selected model school (schools with better faculty and facilities in specific district, 0 = no and 1 = yes) and peer rejection (0 = no and 1 = yes) as measurements for children’s school situations. Residence was classified as living in “1 = rural region” and “0 = urban region”.

### Statistical analyses

Prevalence of unintentional injuries among migrant, left-behind and residential children was presented in the current study. There are two types of dependent variables, one is a dummy variable “suffered unintentional injuries” (0 = no; 1 = yes), the other is a count variable “type numbers of unintentional injuries”. As for the dummy variable “suffered unintentional injuries”, we used Multivariable Logistic Regression Model; while for the count variable “type numbers of unintentional injuries”, which was zero-inflated and over-dispersed, Zero-Inflated Negative Binomial Model was employed in this study [23]. The software STATA 13.0 for windows (Stata Corp, College Station, TX, USA) was utilized for statistical analysis.

## Results

### Sample description

Table 1 shows characteristics of children participants. In a total of 4479 individuals, 23.48% were left-behind children, 34.46% were migrant children, 57.74% were between 6 to11 years old, 46.50% were female, 58.59% had good physical health, 21.61% had good school academic performance and 67.66% were fair, 57.11% were the only-child in family, 45.43% lived in rural area. Approximately, 8.81% investigated children lived in a household with high level income while 62.91% were medium, 91.08% children’s parent were married, 76.83% children’s mother had education of middle school and above, and 93.89% of children seldom or never had family conflicts. A minority of investigated children studied in model school (8.72%) and were rejected by peers (8.07%).

**Table 1:** Descriptive statistics of key independent variables (*N* = 4479)

Variables	Percentage
Type of child (%)	
Residential	42.06
Left-behind	23.48
Migrant	34.46
Individual factors	
Gender (reference: girl) (%)	53.50
Age group (reference: 6–11) (%)	42.26
Health conditions (reference: good physical health) (%)	41.41
School academic achievements (reference: good) (%)	
Fair	67.66
Poor	10.73
The only-child in family (reference: no) (%)	57.11
Parental and household factors	
Household income level (reference: high) (%)	
Medium	62.91
Low	28.28
Parental marital status (reference: unmarried) (%)	91.08
Maternal education attainment (reference: elementary school and below) (%)	76.83
Family conflicts (reference: frequent) (%)	93.89
School situation factors	
Model school (reference: no) (%)	8.72
Peer rejection(reference: no) (%)	8.07
Residence factors	
Rural region (reference: urban region) (%)	45.43

### Prevalence of unintentional injuries among left-behind, migrant and residential children

Table 2 lists the estimated prevalence of unintentional injury among left-behind, migrant and residential children. Overall, the prevalence of unintentional injury was 56.47%. The average number of unintentional injury types was 1.46. The prevalence of falls was 30.44%, ranked as the first among all types of unintentional injuries children suffered from, followed by striking by objects or person with prevalence of 21.90%. Poisoning had the least prevalence (2.52%) among the participants. More details on the prevalence of specific types of unintentional injury are listed in Table 2.

**Table 2:** Bivariate analysis of unintentional injuries among three types of children

	Total	Residential	Left-behind	Migrant	Chi2/F	P
Suffered unintentional injuries(%)	56.47	54.93	64.67	52.75	39.331	0.000
Mean of type numbers of unintentional injuries	1.46	1.38	1.95	1.23	45.720	0.000
Specific kind of child unintentional injury (%)						
Vehicle and traffic injuries	5.26	4.45	7.98	4.40	20.335	0.000
Falls	30.44	30.81	35.42	26.60	23.213	0.000
Struck by thrown, projected or falling objects	4.73	4.29	7.50	3.37	25.145	0.000
Contact with sharp instrument	21.23	19.88	28.87	17.67	50.506	0.000
Striking by objects or person	21.90	22.00	26.50	18.64	22.610	0.000
Caught, crushed, jammed or pinched in or between objects	7.05	6.84	9.50	5.63	14.498	0.001
Explosion and rupture of objects	4.17	3.92	6.65	2.78	23.897	0.000
Foreign body enter	10.30	9.60	13.39	9.06	14.454	0.001
Bitten or struck by animals	10.19	8.96	13.58	9.39	17.430	0.000
Accidental drowning and submersion	3.81	3.55	6.55	2.27	31.985	0.000
Exposure to electric current	3.03	2.81	5.22	1.81	25.321	0.000
Exposure to smoke, fire and flames, contact with heat and hot substances	10.50	9.07	15.10	9.13	30.922	0.000
Injuries caused by nature or environmental factors	11.22	9.70	15.38	10.23	24.227	0.000
Poisoning	2.52	2.60	3.32	1.88	5.417	0.067

Table 2 also presents the bivariate analysis results of prevalence of unintentional injuries by child category. It shows that prevalence of unintentional injury significantly differed between left-behind children and the other two types of children. Left-behind children had the highest prevalence of unintentional injury (64.67%), while migrant children’s prevalence of unintentional injury was similar to residential children. Left-behind children suffered from almost two types of unintentional injuries on average for the past 12 months. Falls ranked the highest prevalence in all child category. Although there was difference in the order of the top five kinds of unintentional injuries, the top five kinds were similar among the three types of children which included falls, contact with sharp instrument, striking by objects or person, bitten or struck by animals and injuries caused by nature or environment factors. It’s worthy to mention that left-behind children had the highest prevalence on each type of unintentional injury. More details on the prevalence of each specific type of unintentional injury among the three categorical children are listed in Table 2.

### Regression results

Table 3 presents the multivariable regression results of child unintentional injury. Nested models were used to estimate the effects of child category, as well as individual, family, school and residence characteristics on unintentional injuries among children aged 6–16. When controlling for other variables, left-behind children were more likely to experience unintentional injuries than residential children (OR = 1.208, *p* < 0.05), which was robust in nested regression models. There was no significant difference between migrant children and residential children on unintentional injury occurrence.

**Table 3:** Regression estimates of unintentional injuries

Variables	Model 1	Model 2	Model 3	Model 4
Child category (reference: residential children)				
Left-behind children	1.502***(0.119)	1.397***(0.113)	1.334***(0.109)	1.208*(0.104)
Migrant children	0.916(0.063)	0.883(0.063)	0.876(0.064)	0.951(0.075)
Individual factors				
Gender (reference: girl)		1.146*(0.071)	1.138*(0.071)	1.127(0.070)
Age (reference: 6–11)		0.935(0.063)	0.934(0.064)	0.936(0.065)
Fair physical health(reference: good)		1.424***(0.089)	1.395***(0.088)	1.360***(0.087)
School academic achievement (reference: good)				
Fair		1.178*(0.088)	1.175*(0.089)	1.162*(0.088)
Poor		1.468***(0.171)	1.468**(0.174)	1.418**(0.169)
The only-child in family (reference: no)		0.878*(0.056)	0.903(0.059)	0.945(0.063)
Parental and household factors				
Household income level (reference: high)				
Medium			0.767*(0.090)	0.818(0.097)
Low			0.666**(0.084)	0.708**(0.090)
Parental marital status (reference: unmarried)			1.008(0.110)	0.990(0.109)
Maternal education attainment (reference: elementary school and below)			0.822*(0.063)	0.841*(0.065)
Family conflicts (reference: frequent)			0.665**(0.093)	0.696**(0.098)
School situation factors				
Model school (reference: no)				0.870(0.096)
Peer rejection(reference: no)				1.576***(0.190)
Residence factors				
Rural region (reference: urban region)				1.288**(0.100)
Constant	1.219***(0.056)	0.955(0.090)	2.177***(0.472)	1.704*(0.382)
N	4479	4479	4479	4479
chi2	39.84	95.06	124.4	153.5
p	0.000	0.000	0.000	0.000

Standard errors in parentheses; * *p* < 0.05, ** *p* < 0.01, *** *p* < 0.001

Children with fair physical health (OR = 1.360, *p* < 0.001) and poor school academic achievements (OR = 1.418, *p* < 0.01) were more likely to suffer from accidental injuries. When controlling for other individual variables, there was no significant difference on gender and age groups of unintentional injury occurrence. Besides, the only-child was not correlated to the outcome measures.

When adjusting for other variables, we found that household circumstances had significant associations with child’s unintentional injury risks. Children living in a family with a more educated mother (OR = 0.841, *p* < 0.05) and less family conflicts (OR = 0.696, *p* < 0.01) were less likely to suffer from unexpected injury. It is worth noting that low level of household income (compared to high household income level) had a significant association with children unintentional injuries (OR = 0.708, *p* < 0.01). No significant difference was found in parents’ marital status on child’s unintentional injury. Holding all other variables constant, it presents that children who were rejected by peers had more possibilities to face unintentional injuries (OR = 1.576, *p* < 0.001). School type was not correlated to the occurrence of child’s unintentional injuries. Notably, children resided at rural areas experienced 28.8% higher level of accidental injury risks than children lived in urban areas (*p* < 0.01).

As can be seen from Table 4, zero-inflated negative binomial regression results show that the associations on the number of unintentional injury types between left-behind and residential children remained statistically significant after adjusting for other variables. We found that left-behind children had 1.12 times (incidence rate ratio, IRR = e^coef.^) than residential children to experience more than one type of unintentional injury (*p* < 0.05). Although the results from Table 2 show that migrant children’s expected numbers for experiencing different types of unintentional injuries were less than residential children, there was no statistical association found in zero-inflated negative binominal regression results.

**Table 4:** Zero-inflated negative binomial of type numbers of unintentional injuries

Variables	Coef.	IRR
Child category (reference: residential children)		
Left-behind children	0.109^*^ (0.051)	1.115* (0.057)
Migrant children	−0.045(0.052)	0.956(0.049)
Individual factors		
Gender(reference: girl)	0.069(0.039)	1.072(0.042)
Age (reference: 6–11)	0.181^***^ (0.040)	1.198^***^ (0.048)
Fair physical health(reference: good)	0.175^***^ (0.040)	1.192^***^ (0.047)
School academic achievements (reference: good)		
Fair	0.116^*^(0.049)	1.123^*^(0.055)
Poor	0.206^**^(0.072)	1.228^**^(0.088)
The only-child in family (reference: no)	−0.028(0.042)	0.972(0.040)
Parental and household factors		
Household income level (reference: high)		
Medium	−0.210^**^(0.068)	0.811^**^(0.055)
Low	−0.242^**^(0.073)	0.785^**^(0.058)
Parental marital status (reference: unmarried)	−0.017(0.067)	0.983(0.066)
Maternal education attainment (reference: elementary school and below)	−0.190^***^(0.046)	0.827^***^(0.038)
Family conflicts (reference: frequent)	−0.288^***^(0.075)	0.750^***^(0.056)
School situation factors		
Model school (reference: no)	−0.121(0.073)	0.886(0.065)
Peer rejection(reference: no)	0.348^***^(0.066)	1.417^***^(0.094)
Residence factors		
Rural region (reference: urban region)	0.304^***^(0.050)	1.355^***^(0.067)
Constant	0.662^***^(0.135)	1.939^***^(0.262)
N	4479	4479
chi2	313.058	313.100
p	0.000	0.000

Standard errors in parentheses; * *p* < 0.05, ** *p* < 0.01, *** *p* < 0.001; both alpha and Vuong statistic tests are significant, and support zero-inflated negative binomial models

Table 4 also indicates that children’s personal characteristics and circumstances they lived in had a strong association with type numbers of child unintentional injury. As for children’s individual factors, while keeping other variables constant, the expected number of unintentional injury types for children aged 12–16 was 19.8% higher than the younger children (*p* < 0.001), those who had fair physical health were 19.2% higher than those with good physical health (*p* < 0.001); children with poor school academic achievements were 22.8% higher than those with good school academic achievements (*p* < 0.01). As for circumstance factors, the expected number of unintentional injury types for children lived in a medium/low level household income were 18.9% (*p* < 0.01) and 21.5% (*p* < 0.01) lower than those in high income family, respectively; children whose mother’s education attainments were middle school and above were 17.3% lower than those with less educated mother on the expected type numbers of unintentional injuries (*p* < 0.001); children lived in a family with less family conflicts were 25% lower than those frequent ones (*p* < 0.001). Children’s peer rejection was statistically associated with the number of unintentional injury types that children experienced. Children being rejected by their classmates had 1.417 times of the expected number of injury types more than the well-integrated (p < 0.001). Children resided in rural region experienced 35.5% higher expected number of injury types than those in urban region (*p* < 0.001).

In short, this study shows that left-behind children had higher risks on experiencing unintentional injury and left-behind children had higher probability to suffer from different types of injuries than residential children, but we found no difference between migrant children and residential children; both individual characteristics and circumstance factors were related to children unintentional injuries among the children.

## Discussion

### The effects of migration

First, this study examines the difference of child unintentional injury prevalence among three children groups, which may partly reveal the potential impact of migration on children. The prevalence of unintentional injury was 64.67% among left-behind and 52.75% among migrant children in this study. The prevalence of unintentional injury among left-behind children or migrant children in former studies were reported with the range of 19.4%–64.3% [24]. However, we found that the prevalence of left-behind and migrant children reported in previous studies were not comparable for the following reasons. First, the age groups of children being measured and the places for survey varied in different literatures; second, most researches were not conducted in accordance with the definition and classification of unintentional injuries of ICD-10; third, the information of child’s unintentional injury was answered by children rather than guardians in some researches which may result in underestimation. Besides, most of these studies were in small sample size. As there is no official statistical prevalence on unintentional injury of left-behind and migrant children in school age, we argue that the prevalence on unintentional injury of left-behind and migrant children estimated in this study was reliable, based on the reasons that larger sample size, application of ICD-10 and restricted investigation procedure conducted in this survey.

For the types of unintentional injury, falls, sharp or blunt force and animal bites were ranked as the major types for the three categorical children, which was consistent with previous studies [25]. This finding suggested that the three types of children faced with similar injury risks for both indoor and outdoor accidental environments.

Second, this study suggests that left-behind children were more vulnerable to experience unintentional injuries than residential children, which was consistent with previous studies [26]. Besides, the average number of unintentional injury types that left-behind children suffered from was 1.95 while the mean was 1.46 for all participants, indicating that left-behind children were much likely to being exposed to environments with various unintentional injury risks. Being left-behind means the absence of parental care resulting from one or both parents’ migration. Children live without parental care may lack of security guidance and protection from unintentional injury, which may increase the probability to suffer from injuries, especially for those who are guarded by their grandparents or other family members due to both parents’ migration [27].

However, this study found no significant difference on unintentional injury prevalence between migrant and residential children, which was contrary to our original hypothesis. Although some previous studies showed that migrant children had higher risks on unintentional injury than residential children, some argued that no significant difference existed in both types of children [28]. Most of the migrant children in China follow their parents to move from rural areas to urban areas. Migration means a change of original living surroundings and adaption to new environments for children. However, migrant children live with one or both parents, enabling them to live with parental supervision, as well as to acquire prevention from unintentional injury. In terms of parental care, migrant children do not have significant difference with residential children, but have better parenting conditions than left-behind children, especially for those with both parents’ migration.

Based on findings mentioned above, we suggest that there was profound meaning on the difference of unintentional injury among the three types of children. On the issue of child’s unintentional injury, the absence of parental care has more negative effects on children than the changes of living surroundings. Different types of family migration exert various impacts on children, making them face with diverse challenges and vulnerabilities. Specific attentions should be paid to unique groups of children.

Our study also shows that children’s unintentional injury outcomes differed by their personal characteristics. Children aged from 12 to 16 had higher risks for experiencing various types of unintentional injuries than the younger, suggesting that the elder children, most of whom were middle school students, may expand their access to the environments, such as homes, schools and other open air places which increase the potential injury hazards than the primary school children [29]. In accordance with previous studies, our findings suggested that children with good school academic achievements were less likely to experience unintentional injuries than those with fair or poor performances [30]. This may be because children with better school achievements tend to have a sense of self-protection and self-control, as well as to acquire knowledge on security which may improve their attitudes and practices towards unsafe behaviors.

### The effects of family environment

Our study shows that household socioeconomic factors were strongly associated with children unintentional injuries. Children with less educated mother were in risk of unintentional injury which was consistent with previous studies [31]. The possible reason may be that a better educated mother tends to be equipped with better knowledge and ability on children injury prevention. In controversy, low educated mother may be more likely to lack of safety consciousness and parenting skills, as well as knowledge and ability on child protection.

Although previous studies suggested that family poverty was a major risk factor for unintentional injury [32], our findings notably suggested that after adjustments for other individual and environmental variables, children lived in a lower income household were less likely to experience unexpected injuries, which is in line with Wen’s study [33]. This may be because children living in higher income households seem to be more social participated, active and have more access to expand their scopes of activities which may increase the potential injury hazards. Our survey data also showed that children in a medium and high income household were more active and energetic than those live in low income households, 81.98% of children from richer household had no trouble being exposed to public occasions while only 70.68% for the poor; furthermore, 88.61% of children from high income household behaved actively in public but 77.37% for the poor. Therefore, children live in high income households may experience higher risks of unintentional injury due to more access to risk occasions and more active personality.

Harmonious family relationship is an important protective factor in children’s exposure to unintentional injury. A household with less conflicts provides children a stable and harmonious family environment against injury risks [34]. Previous studies had debates on the association between number of siblings and child unintentional injury risks [35, 36]. Our findings suggested that there were no statistically difference on only-child and non only-child in unintentional injury risks. Due to the family planning policy in China, most families have no more than two children which makes children being regarded as family treasures. Thus, family may not reduce their parenting care whether the child is the only-child or not.

### The effects of school environment

We found that good peer relationship benefited for preventing unintentional injuries. School is one of the most frequent environments that children experience unintentional injuries and social support from peers or classmates may provide useful information and protection to some extent against unintentional injury risks [17].

There was no higher or lower unintentional injury risk for children whether studying in model schools. Some previous studies showed that model schools had more resources and better management system on reducing the risks of child unintentional injury [37]. On the other hand, model schools are equipped with more facilities and usually located in crowded areas which may increase risks for child unintentional injury.

### The effects of residential environment

We found that residence was associated with child unintentional injury, indicating that children living in rural areas had more odds than their urban peers to experience unintentional injury, which was in line with previous studies. Studies showed that living and housing surrounding contexts, such as the traffic volume, absence of safe recreational spaces and less access to childcare facilities, may increase the child injury risks. In addition, the underlying urban-rural difference in lifestyles, attitude to risk behaviors potentially lead to different consequences on injury [38].

This study has a few limitations. First, the frequency and severity for unintentional injuries were not included in the survey. Therefore, we were unable to measure the severity of unintentional injuries. Second, a cross-sectional design for this study cannot draw a causal association of personal characteristics and circumstance factors with the outcome measures. Third, given the sensitive and serious nature of child unintentional injury, it was possible for parents or guardians to offer desirable answers which may cause bias. However, compared with reports from teachers and children themselves, information reported by parents or guardians may be the most appropriate and reliable. Besides, some efforts (i.e., detailed explanations for specific questions, keeping parents or guardians from interruption) had been done by the investigators to improve the survey quality and to reduce the possible bias.

Several strengths of the current study, to some extent, balance its limitations. First and foremost, we applied measurement of unintentional injury in accordance with the definition and classification of ICD-10 which was more comprehensive and scientific. Second, a school-based survey designed with population representativeness for this study can capture the information of all types of children. Third, this is the first comparative study on unintentional injury among left-behind, migrant and residential children in China, and represents one of the most comprehensive studies on this issue in developing nations with large scale of migration. Last but not the least, we measured not only individual factors but also environmental factors which was more hierarchical based on the ecological theoretical framework.

Based on the findings of this study, more attention and resources should be allocated to protect children from unintentional injuries, and more practical approaches should be employed, especially for the left-behind children. It’s advisable for local governments to establish an information database for left-behind children with dynamic management to enhance the regulatory capacity of unintentional injuries. As well, it would be helpful to construct more safe recreational spaces for left-behind children and to improve more access to childcare facilities. With regard to schools, targeted courses are expected to be developed to raise left-behind children’s self-protection awareness. On the other hand, teachers are required to focus more on left-behind children’s physical and mental health. Significantly, frequent communication among local governments, schools and guardians is of great importance for creating a positive atmosphere to care for left-behind children.

## Conclusion

This study shows a higher prevalence of total unintentional injury and in each specific type of unintentional injuries in left-behind children than migrant and residential children. Personal characteristics including age, physical health and school academic achievements, environmental factors such as household income, maternal education attainment, family and peer relationships as well as residence, were significantly associated with child unintentional injury. Family migration exerts significant impacts on child unintentional injury risks, more attention should be paid to parenting care and living surroundings for left-behind children and migrant children. Special attention ought to be paid to children suffering from unintentional injuries, especially for those who experienced family migration. More targeted measures are encouraged to provide children with well-equipped facilities, to enhance children’s awareness of self-protection, and to create an atmosphere for the whole society to focus on the unintentional injuries of children.
